# The role of noise and positive feedback in the onset of autosomal dominant diseases

**DOI:** 10.1186/1752-0509-4-93

**Published:** 2010-06-29

**Authors:** William J Bosl, Rong Li

**Affiliations:** 1 Harvard Medical School, Boston, MA 02115, USA; 2 Children's Hospital Informatics Program at Harvard-MIT Division of Health Sciences and Technology, Boston, MA 02115, USA; 3 Stowers Institute for Medical Research, Kansas City, MO 64110, USA

## Abstract

**Background:**

Autosomal dominant (AD) diseases result when a single mutant or non-functioning gene is present on an autosomal chromosome. These diseases often do not emerge at birth. There are presently two prevailing theories explaining the expression of AD diseases. One explanation originates from the Knudson two-hit theory of hereditary cancers, where loss of heterozygosity or occurrence of somatic mutations impairs the function of the wild-type copy. While these somatic second hits may be sufficient for stable disease states, it is often difficult to determine if their occurrence necessarily marks the initiation of disease progression. A more direct consequence of a heterozygous genetic background is haploinsufficiency, referring to a lack of sufficient gene function due to reduced wild-type gene copy number; however, haploinsufficiency can involve a variety of additional mechanisms, such as noise in gene expression or protein levels, injury and second hit mutations in other genes. In this study, we explore the possible contribution to the onset of autosomal dominant diseases from intrinsic factors, such as those determined by the structure of the molecular networks governing normal cellular physiology.

**Results:**

First, simple models of single gene insufficiency using the positive feedback loops that may be derived from a three-component network were studied by computer simulation using Bionet software. The network structure is shown to affect the dynamics considerably; some networks are relatively stable even when large stochastic variations in are present, while others exhibit switch-like dynamics. In the latter cases, once the network switches over to the disease state it remains in that state permanently. Model pathways for two autosomal dominant diseases, AD polycystic kidney disease and mature onset diabetes of youth (MODY) were simulated and the results are compared to known disease characteristics.

**Conclusions:**

By identifying the intrinsic mechanisms involved in the onset of AD diseases, it may be possible to better assess risk factors as well as lead to potential new drug targets. To illustrate the applicability of this study of pathway dynamics, we simulated the primary pathways involved in two autosomal dominant diseases, Polycystic Kidney Disease (PKD) and mature onset diabetes of youth (MODY). Simulations demonstrate that some of the primary disease characteristics are consistent with the positive feedback - stochastic variation theory presented here. This has implications for new drug targets to control these diseases by blocking the positive feedback loop in the relevant pathways.

## Background

Many human genetic diseases result from loss-of-function germline mutations in one of the two homologous gene loci. These are often referred to as autosomal dominant diseases because of frequent phenotypic dominance of the mutated allele over the wild-type allele during transmission along generations. There are presently two prevailing theories explaining the autosomal dominant expression of these diseases. One explanation originates from the Knudson two-hit theory of hereditary cancers, where loss of heterozygosity or occurrence of somatic mutations impairs the function of the wild-type copy [1-3]. While these somatic second hits may be sufficient for stable disease states, it is often difficult to determine if their occurrence necessarily marks the initiation of disease progression [4,5]. A more direct consequence of a heterozygous genetic background is haploinsufficiency, referring to a lack of sufficient gene function due to reduced wild-type gene copy number; however, haploinsufficiency can involve a variety of additional mechanisms, such as noise in gene expression or protein levels, injury and second hit mutations in other genes [6]. These stochastic factors are likely to contribute to the characteristics of variable time of onset and incomplete penetrance of many autosomal dominant diseases [7]. In this study, we explore the possible contribution to the onset of these diseases from intrinsic factors, such as those determined by the structure of the molecular networks governing normal cellular physiology.

Cook (1998) proposed that stochastic gene expression might play a role in haploinsufficiency disease. The primary mechanism suggested was the stochastic fluctuation of gene expression that could cause an essential gene product to fall below some critical level. Since then a number of papers have supported this theory by quantifying variability in gene expression [8-13]. One potential weakness of this theory is that the disease condition should be expected to improve or disappear when the gene product levels return to normal or the mean level [14]. In order to achieve a stable disease state, an additional switching mechanism may be involved. A common switching mechanism found in biological systems is a positive feedback loop structure.

Positive feedback loops are network structures that appear to have two functions in biological systems: they act as rapid switches to turn on a process [15,16] and they act as noise buffers to enable a system to respond to long term signal changes while resisting the effects of transient fluctuations [16]. Because of this dual ability, it was suggested that positive feedback loops impart an evolutionary advantage as a biological switch mechanism. However, positive feedback loops that balance both these properties are difficult to achieve because such positive feedback systems may depend on having production rates of key components that are high enough to overcome the effects of noise or environmental perturbations and yet low enough to maintain sensitivity. When one allele of a critical gene is lost, as in the case of autosomal dominant disease background, the stability of the noise-resistant, rapid-on switch may be compromised.

Experiments with small networks of neurons as well as simulations have shown that networks composed of both excitatory and inhibitory elements can exhibit complex behavior [17,18]. One of the principle characteristics of a complex system is sensitive dependence on initial conditions [19,20]. Small perturbations to the system may cause large changes in the system, including rapid changes to new states; in other cases, large perturbations may cause little change to the system. A network composed of positive feedback loops and a combination of activation and inhibition may exhibit complex system characteristics.

Two important sources of noise in biological networks are gene expression fluctuations on several time scales and environmental perturbations due to such events as injury or disease. Variability in protein levels occurs on a range of time scales, from minutes to many cell lifetimes to permanent (Sigal, et al., 2006). On the most rapid scale, protein levels were found to fluctuate on time scales of 15 to 50 hours or 0.8 to about 2.5 cell cycles. Fluctuations were highly correlated between proteins in the same pathway, but not between different pathways, implying that most of the variability is due to upstream regulatory components in specific pathways. Stochastic gene expression noise has been explained by several different mechanisms [21,22]. When two alleles are functioning properly, random fluctuations in gene expression may play a positive role in cellular networks and are thought to be important for cell differentiation and autostabilization [23,24]. Stochastic gene expression might also provide a simple mechanism for organisms to explore genetic design space without having to go through the all-or-none drastic step of gene mutation. Environmental noise caused by injury or disease is more difficult to characterize than the noise in gene expression, in part because it can result from many different causes. Nevertheless, perturbations external to cells can have a marked temporary effect on cellular networks that can be prolonged by certain network structures.

In this paper, we explore the hypothesis that the combination of network noise together with positive feedback loops results in dynamic behavior that can explain the etiology of some autosomal dominant diseases. A number of positive feedback network structures are examined using modeling to simulate the dynamics when the expression level of key components is reduced by one half and subjected to Gaussian noise to simulate stochastic gene expression. Key factors that affect network response are the time scale of random fluctuations, amplitude of the variation, and the structure of the feedback loop.

All network simulations were done using Bionet software, which is available through the Stanford Simbios National Center for Biomedical Computing website [25]. The fuzzy logic methodology in Bionet is common in the design and modelling of advanced control systems in many engineering disciplines. This approach was adopted for pathway modeling to allow experimental biologists to use the kind of qualitative data found in typical journal articles to describe the interaction of genes, proteins, and other cellular components to create computer models of large numbers of interacting components. Fuzzy network modeling can be used as a tool for aiding human reasoning when many interacting variables participate in complex interaction networks on several scales. Though the interactions can sometimes only be described approximately, the logic of the interactions is rigorous. Importantly, Bionet simulates network dynamics as accurately as differential equation models, but without explicitly defining equations. Detailed information about the modeling methodology in Bionet can be found in [26].

The goal of this study is to examine the effect of haploinsufficiency on network dynamics and to illustrate that the combination of stochastic gene expression and certain network structures may explain some autosomal dominant disease characteristics. Parameters that represent gene expression levels in the following simulations must be set so that when 2 genes are functional, the network is stable. But if the expression levels are set too high, then disabling one gene will never have any effect. The expression level of one gene should be set so that the network is still stable if there is no noise in the expression. Otherwise, since the network dynamics is deterministic when no noise is present, the network will always fall into one final state immediately. The characteristic of autosomal dominant diseases that is to be simulated here is onset that does not occur immediately, but exhibits some variation in the age of onset. This can only be achieved when the expression parameters are in a relatively narrow range. Furthermore, only one gene is heterozygous at a time. The normal, homozygous genes both express at normal levels and thus variations in their levels will not degrade the network performance. These simulation conditions reflect the hypothesis to be tested here: haploinsufficiency of a single gene in the key pathway for an autosomal dominant disease may affect the disease state for certain network structures.

## Results

### Three component positive feedback loop simulations

**Figure 1:**
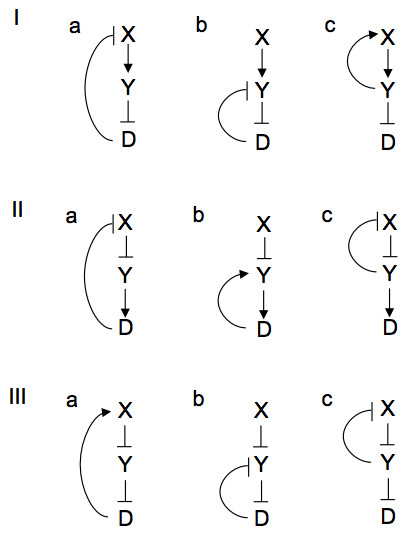
**Positive feedback loops**. All possible configurations of positive feedback loops that can be derived from a three component network.

**Figure 2:**
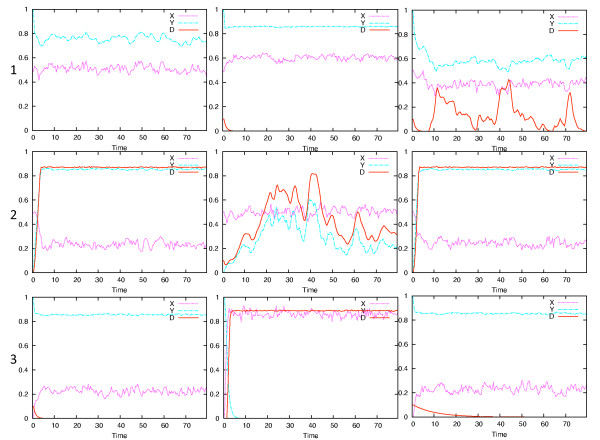
**X haploinsufficiency**. Simulation results for each of the networks in Figure 1 when X is haploinsufficient and Gaussian noise is added to X production.

**Figure 3:**
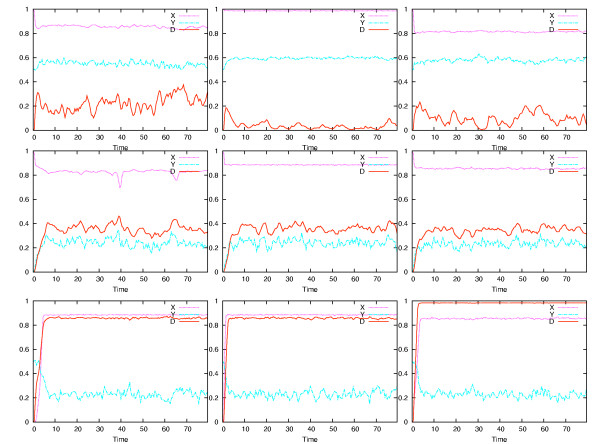
**Y haploinsufficiency**. Simulation results for each of the networks in Figure 1 when Y is haploinsufficient and Gaussian noise is added to X production.

All of the possible positive feedback loops that may be derived from a three-component network are shown in Figure 1. A positive feedback loop is defined here as a loop that encompasses at least two nodes and has an even number of negative regulators, either zero or two, in the loop. In each case, X is the signalling protein at the top of the loop, Y an intermediate protein, and D is a marker for the disease state. Simulations were run with either X or Y production reduced by one half, as a consequence of heterozygosity, and Gaussian noised added to the expression of the affected gene product. Time steps of one half week, and thus random variation on the same time scale, were similar to the lower end of random variations reported earlier [21]. Figure 2 shows time courses over an 80-year period for all of the loops in the X-heterozygosity case and Figure 3 shows the Y-heterozygosity cases. This time span was chosen to approximately represent a normal human lifespan. Network structures that cause D to increase to high levels early and irreversibly may be characterized as allowing or causing a high level of disease symptoms. These include 2.a, 2.c and 3.b for X-heterozygosity and all of the type 3 loops for Y-heterozygosity. Milder or variable disease symptoms may be expected when the D time course is somewhat repressed or arises intermittently. These include 1.c and 2.b for X- heterozygosity and 1.a, 1.c, all type 2 loops for Y heterozygosity. No disease occurs in networks 1.a, 1.b, 3.a and 3.c for X- heterozygosity; no such cases occur for Y-heterozygosity, although 1.b has a very low level of variable disease presence. These results are summarized in Table 1. The characterization of network dynamics in terms of disease onset as mild is somewhat general, with a wide range of dynamic responses possible. The severe labels (++) indicate cases where disease onset would be expected to be early, symptoms relatively stable and the likelihood of occurrence almost certain.

**Table 1:** Noise and haploinsufficiency in different loop structures

Network Type	Network Type	a	b	c
1	X deficient Y deficient	0 +	0 -	+ +
2	X deficient Y deficient	++ +	- +	++ +
3	X deficient Y deficient	0 ++	++ ++	0 ++

Effect of Gaussian noise and haploinsufficiency on disease dynamics with different positive feedback loop structures. ++ indicates early, irreversible and severe disease onset; + indicates disease onset is variable and slightly suppressed; - are cases where disease fluctuates randomly, but reversibly and is suppressed as soon as the appropriate protein rises or falls; 0 indicates no disease occurs.

### Sensitivity to Noise

Sensitivity of the networks examined above to parameter changes is a key issue. The critical parameter in this study is the amount of variation in the haploinsufficient gene, which is equivalent to the production rate of the relevant protein. Note that 'production rate' in our models is synonymous with 'expression level'. In all of the networks, including those for specific diseases discussed below, the behavior of the network is completely determined and stable when expression levels are constant. Furthermore, in order to simulate the biological conditions of interest, the expression level of the network elements is such that when two genes are functioning, the network is unconditionally stable. That is, the production rate is high enough that the network remains in the disease-free state even in the presence of noise or variation in rate. If a single gene mutation occurs so that only one gene is operative, the production rate of that gene cannot be so high that it effectively eliminates the haploinsufficiency condition. If the expression levels are set so high or low that the random variation is irrelevant, then, again, the final steady state of the system will be completely determined from the start: the disease will either be present always or never. Therefore, the parameters that control expression levels in this study are necessarily set near critical points for single genes. The assumption underlying this entire study is that two genes must express sufficiently for a stable, non-disease state to exist even when the expression levels vary stochastically, while a single gene is such that the disease state sometimes occurs, sometimes does not. This is the characteristic of autosomal dominant disease that the simulations seek to reproduce and explain.

The critical parameter that is relevant to autosomal dominant disease in the simulations presented here is the magnitude of the concentration variation of the relevant protein. The protein concentration varies because of random expression variation of the deficient geneThat is, the rate of production varies, but the concentration of the product is what is relevant to the disease state. Some of the network types exhibit a linear and reversible dependence of the disease state on the magnitude of stochastic variation in the haploinsufficient gene. In Figure 2, 1c and 2b exhibit this behavior and in Figure 3 all of types 1 and 2 exhibit this behavior. Even if the relevant deficient gene expression level temporarily drops to zero, the disease state, represented by D, decreases again when gene expression rises.

**Figure 4:**
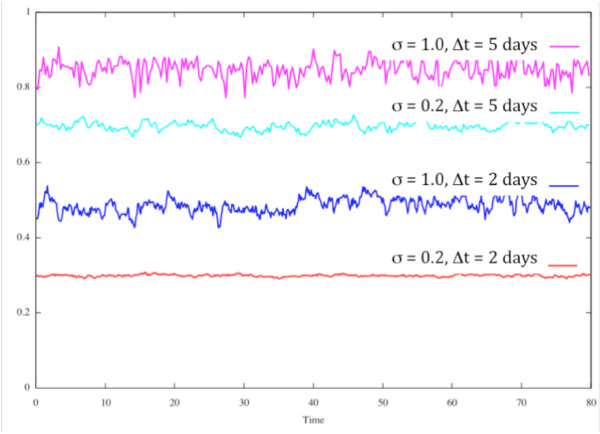
**Effect of Gaussian noise added to production rate on product concentration**. Gaussian noise added to the production rate causes changes in the concentration of the protein. The standard deviation of the noise, σ, and the size of the time step over which the rate is changing, Δ, both affect the size of the concentration variations as shown here.

The variation in protein concentration due to adding a Gaussian noise term to the production rate is illustrated in Figure 4. The curves are offset for visual illustration. Each curve represents a protein concentration that is maintained by a constant decay rate and a production rate that consists of a constant plus a Gaussian random noise term. The noise in each of these cases has a standard deviation of either 1.0 or 0.2. Since the concentration is affected by how long the production rate term deviates from its average value, the time step over which the random variation is changing also affects the concentration variability. It is clear in Figure 4 that a longer time for production rate deviation results in larger variability in product concentration.

**Figure 5:**
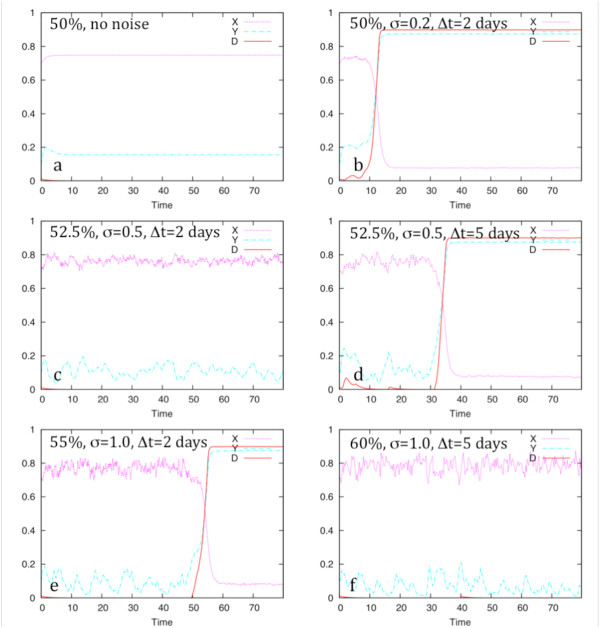
**Illustration of network response to changes in stochastic parameters**. The response of network 2a to variations in stochasticity when X is deficient is used here to illustrate the effect of changing the noise added to production rate on the product concentration. These are single snapshots of the 100-simulation trials shown in table 2. Note that the normalized protein concentration is plotted, while the expression level refers to the rate of protein production; the rate is not plotted.

When the disease state is involved in the feedback loop as in Figure 2a, a natural instability exists. The two stable states of interest are the presence or absence of disease. In all cases the initial state is assumed to be the absence of disease; D is initially zero. This prevents the situation where D is initially high and is involved in a feedback loop that completely blocks a switch to a low D state and is biologically realistic because haploinsufficiency diseases do not often exhibit the disease state immediately and deterministically. In network type 2.a, if X is initially set above a critical level, X_c0_, and there is no stochastic variability, D will remain near zero, as shown in Figure 5a. Similarly, if X is set below X_c0_, D will rise to its highest level and remain there. A plot of the latter case will be very similar to Figure 5b.

The response of network 2a to variations in stochasticity when X is deficient is an example of a network with two stable states, disease-free and disease. In order to study network behavior with different levels of the noise parameter, 100-simulation trials were run and the final state of the system was determined. Single snapshots of the 100-simulation trials are shown in Figure 5 and the statistical results in Table 2. a. When no noise is present in X, the system dynamics are deterministic. Expression levels are defined and set as follows. The critical expression level for the gene X is determined empirically through computational experimentation. This is the basal expression level for a single gene in this sensitivity study. Define the normal expression level to be when two genes each express at the critical level. This will be considered 100% expression or normal expression. Haploinsufficiency occurs when one gene is inactive and the expression level is expected to be 50% of normal. In Figure 5, 50% refers to a single gene expressing or producing protein X at a rate that prevents D from rising in the deterministic case (no noise in the expression level). If the expression level is lowered by a small amount, for example 49%, D will rise. Note that the graphs in Figure 5 show protein levels, but the stochastic variation is in the rate of protein production. The expression levels, such as 50%, refer to the production rate of the protein, not the actual concentration that is plotted. The basal rate is constant throughout the simulation, with Gaussian noise added to the rate.

**Table 2:** Sensitivity of feedback loop to changes in stochasticity parameters

Trial	Expression level	Noise stdev	Time scale (days)	Penetrance	Mean onset
1^b^	50%	0.2	2	0.31	2.1/5.9
2	50%	0.5	2	0.94	22.1/20.4
3	50%	1.0	2	1.0	12.5/10.6
4	50%	0.2	5	0.91	21.9/21.8
5^c^	52.5%	0.5	2	0.20	6.1/18.7
6	52.5%	1.0	2	0.95	17.8/15.9
7^d^	52.5%	0.5	5	0.54	7.5/13.0
8	52.5%	1.0	5	1.0	22.0/18.5
9	55%	0.5	2	0.0	--
10	55%	0.5	5	0.0	--
11^e^	55%	1.0	2	0.34	8.8/19.2
12	55%	1.0	5	1.0	2.7/0.1
13^f^	60%	1.0	5	0.0	--

Sensitivity of feedback loop 2.a to changes in stochasticity is illustrated here using 100-trial simulations. Expression level is such that 50% is at the critical level: slightly lower expression, with no noise, allows disease to deterministically occur all the time. 100 simulations for each set of conditions were run to compute a penetrance value. When no noise is present, the system is deterministic and no disease occurs as long as the base expression level is 50% or higher. When expression is 60% or higher, no disease occurs even with the highest level of random variation used in this study.

The stochastic variation in all simulations in this study is determined by adding a zero-mean Gaussian value to the basal expression rate. The standard deviation of the Gaussian term is denoted by σ. Thus, (0, 0.5) noise refers to a Gaussian distribution centered on zero with a standard deviation of 0.5. In the simulations, a single value is chosen from this distribution at each time step and added to the basal production rate. Since the random variable can be either positive or negative (since the mean of the random variables is zero), the expression rate at any time step is above or below the basal value. When time steps are longer, the relative time that the rate is above or below the basal value is longer and the actual protein concentration drifts farther from its equilibrium value. Thus larger time steps with the same random distribution cause greater variation in the protein concentration.

As can be seen in Figure 5a, the network is stable with one gene when there is no noise. The remaining cases in Figure 5 illustrate the effect of both raising the basal level of expression of the single functioning gene and changing stochastic variation. For the random variation, two parameters are varied. Gaussian noise is added to the expression rate, not the actual value of the protein concentration. The time parameter shown is the length of time over which the expression rate varies. If the time variation is small, the actual protein concentration varies little. As the time for stochastic variation increases, there is greater chance of the key protein, X, drifting via a random walk far enough from the critical value to cause the disease state to increase. Positive feedback then enables a switch to the full disease state. As the basal expression level increases, the penetrance of the disease state decreases. At 60% of normal expression (Figure 5f), the largest noise level used in this study does not allow disease to grow in any trials.

Summarizing the results described above, for a model of autosomal dominant disease based on stochastic variation and positive feedback network structures, several key parameter values are required. First, the expression level of a single gene has to be near a critical point. If the expression level of a single gene is too high or low, the disease will either never be present or always be present from birth. Second, the stochastic variation in expression level must have a large enough deviation from the mean to cause the concentration of the key protein concentration to dip low enough to trigger positive feedback from the disease state. But this alone is insufficient; the length of time of the variations in expression rate must be long enough to affect the concentration of the protein. Concentrations do not respond immediately to a drop in expression, but depend on the rate of protein decay.

The following disease models are not intended to determine the precise stochastic parameters that are operative in each of the pathways presented, rather to illustrate with reasonable parameters that the presented disease mechanism is plausible and should be investigated further.

### Polycystic Kidney Disease

Autosomal Dominant Polycystic Kidney Disease (ADPKD) is a common hereditary disorder characterized by cystic dilation of the kidney tubules, eventually leading to enlarged cysts isolated from their nephrons [27]. The resultant decrease in kidney function often necessitates hemodialysis or organ transplantation. The majority of ADPKD (85-90%) are caused by mutations within *PKD1*, encoding a 460 kDa G protein-coupled receptor known as polycystin-1 (PC1) [28,29]. Mutations in a second gene, *PKD2*, abrogate the function of the Ca^2+^-permeable cation channel protein, polycystin-2 (PC2) and account for 10-15% of ADPKD [30-33].

The autosomal dominance of ADPKD refers to the fact that patients carrying a heterozygous mutation in one of the PKD genes have a high chance of disease in their lifetime. However, there is no evidence that the proteins encoded by the mutant alleles acts dominantly over the wild-type allele product. In mouse, heterozygous inactivation of one of the PKD genes also leads to development of kidney cysts in 20-40% of the animals [34]. The main hypothesis in the field currently proposes that disease onset in ADPKD is triggered by somatic "second hit" mutations that inactivate the functional copy of the PKD gene followed by colonal expansion of the affected cells [35]. However, other studies indicate that polycystins are expressed in most cyst lining cells, and even "wild-type" cells can contribute, to a large extent, to cyst growth in the presence of PKD1 mutant cells in chimeric mice, suggesting that somatic mutations may not be the only mechanism to induced ADPKD [36-40].

**Figure 6:**
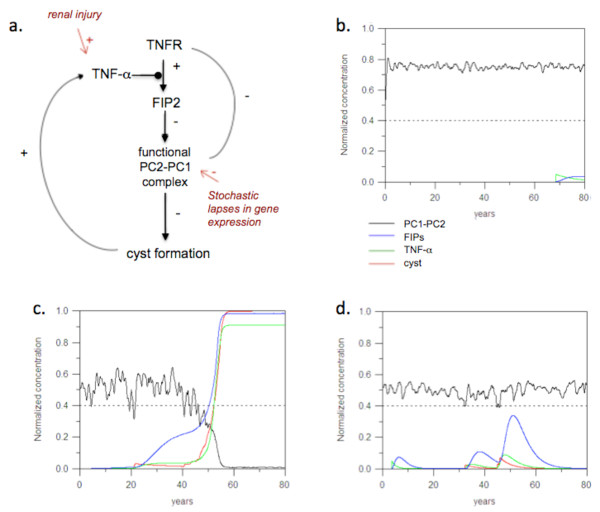
**TNF-α-mediated feedback loops in the control of ADPKD onset**. a. Pathway diagram of the proteins and network involved in cytogenesis. b. Simulation of time courses of primary network components in the wild-type case. Random (Gaussian) noise is added to the expression level of each polycystin allele. Random fluctuations are damped because the expression noise in the two genes is uncorrelated. c. Heterogeneous background simulation. The level of polycystin is lower and the fluctuations are higher. d. Heterogenous background simulation, but with the TNF-α feedback loop disabled. Random fluctuations allow cyst formation to occur, but these are suppressed as soon as above-threshold expression of TNF-a is restored.

Alternative to genetic changes, it is possible that some environmental or non-genetic factors, probably those embedded in the cellular networks involving polycystins, promote the dominant phenotypic expression of ADPKD. In a recent study [41], it was shown that the inflammatory cytokine, TNF-α, negatively modulates the function of PC2 and promotes cyst formation in Pkd2+/- mice. PC2, in turn, negatively regulates the level of TNF-α converting enzyme (TACE) and TNF-α receptor. An increase in TNF-α in renal tissues can also be caused by injury or infection or by cystic conditions as the cytokine was found to accumulate in cyst fluid from human ADPKD patients. These interactions form a network that connects cytokine, polycystin and cystic disease through two feedback loops belonging to loop types 3a and 3c (Figure 6a). Both of these feedback loops could potentially induce a stable disease state if a polycystin (positioned as Y in the feedback loops) is affected by heterozygosity.

Computer simulation with the Bionet program [26] were developed to test the potential role of the above network in the onset of cystogensis associated with ADPKD. PC1 and PC2 are modeled as a functional unit, the level of which is affected by gene dosage of PKD1 and PKD2, as well as proper targeting of PC2 to the cilia, which is inhibited by TNF-α (through induction of a protein called FIP2). There are two sources of random fluctuations in the model: stochastic gene expression and renal injury. The latter occurs infrequently but is sufficient to generate transient spikes in TNF-α production. In the wild-type population, the fluctuation in functional PC1-PC2 level is less in magnitude than in the heterozygous background and is always above the functional threshold required for preventing cyst initiation (Figure 6b). However, in the heterozygous background, where the level of expression is in general sufficient to suppress cyst formation, random fluctuations may cause the feedback loops to be switched on and cyst growth continues unabated (Figure 6c, a random simulation example is shown). This result suggest that a simple network structure could mimic the effect of somatic mutations in disease induction, but the difference is that in the former case, if the TNF-α feedback loop is disabled, full-scale cystogenesis can be prevented even with stochastic sub-threshold dips in the polycystin level (Figure 6d). Indeed, in a mouse ADPKD model, blocking TNF-α signaling inhibited cyst formation [41].

### Maturity onset diabetes of the young

Maturity onset diabetes of the young (MODY) is a clinically heterogeneous group of disorders characterized by an autosomal -dominant mode of inheritance [42]. The diagnosis of MODY can be differentiated from type 2 diabetes in young patients by family history and the presence (or a lack) of obesity. Patients may have mild hyperglycemia, while others have varying degrees of glucose intolerance before developing persistent hyperglycemia. Onset is usually before age 25 and often in childhood or adolescence [42]. In some cases the onset of symptoms may be rapid. Symptoms may be mild to more severe, but in all forms of MODY the problem is insulin deficiency due to a defect in pancreatic b-cell function rather than a defect in insulin action [43]. Mild hyperglycemia may be present for years before symptoms become recognizable and a diagnosis is made, typically between 10 and 30 years of age. In some cases, progression from asymptomatic to hypoglycemia requiring oral medication or insulin may happen quickly [43]. Unfortunately, even mild but untreated elevated blood sugars may cause injury to organs over many years, causing such conditions as neuropathy, retinopathy, renal disease, heart disease and the other detrimental complications of diabetes.

The primary types of MODY, the responsible gene deficiencies and typical treatment (indicative of severity) are shown in Table 3. One of the genes encodes the glycolytic enzyme glucokinase (associated with MODY2) and the others encode transcription factors. MODY types 1 and 3 are caused by mutations to the hepatocyte nuclear factors, hnf-4a and hnf-1a respectively [43,44]. Though MODY3 is more common than MODY1, the mechanism of action and phenotypes are similar. With both of these types, patients may respond to oral drugs early in the disease course, but over time fail to respond to these drugs and symptoms worsen [42-44]. MODY2 is caused by loss of a single allele of the glycokinase gene and tends to have milder symptoms that can often be controlled by diet and exercise alone. MODY types 4 and 5 are rare; MODY4 usually has mild symptoms [45], while MODY5 tends to have more severe symptoms, requiring treatment with oral hypoglycemic agents or insulin [44].

**Table 3:** MODY characteristics

MODY type	Gene deficiency	**Prevalence**^**(1)**^	**Treatment**^**(1,2)**^	Simulated mean/std of onset age	Simulated mean/std insulin
1	Hnf-4α	Uncommon	Oral medication or insulin	10.3/1.2	0.18/0.02
2	glycokinase	Common	Diet and exercise	4.5/0.3	0.26/0.005
3	Hnf-1α	Most common	Oral medication or insulin	24.0/8.1	0.24/0.08
4	Pdx1	Rare	Oral medication or insulin	22.6/11.0	0.22/0.11
5	Hnf-1β	Rare	Insulin	9.3/0.4	0.08/0.004

Observed and simulated characteristics of the five major MODY types. The absolute magnitude of the simulated values is not as important as the relative values. The standard deviation in the age of onset is much larger for MODY3 and MODY4, which should be reflected in a range of onset ages in epidemiological data. Mean and standard deviation of the insulin levels is related to the severity of the disease. Data for "Prevalence" from [42,44].

**Figure 7:**
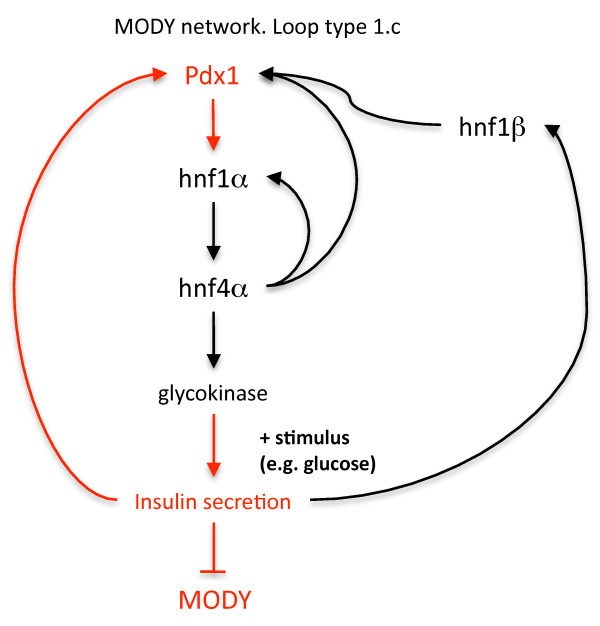
**Key pathway involved in MODY diabetes**. The pathway components highlighted in red illustrate that the basic network structure is of type 1.c, with a subnet of type 1.c embedded in a larger network of the same type. The disease type that results from haploinsufficiency of each gene is shown in parentheses.

The key pathway involved in MODY onset is shown in Figure 7. This pathway contains a network structure of type 1c, but the dynamics are complicated by another embedded sub-network of type 1c and extra feedback loops. As seen in Figure 2, networks of type 1c are more sensitive to deficiencies in X than in Y (Figure 3). The implication is that an upstream component will have a more significant impact on the network dynamics when a deficiency in production exists.

To gain some understanding of the effect of the feedback loops on system dynamics, simulations of the MODY network were carried out with the Bionet modeling program [26]. To generate results for statistical analysis, 100 80-year simulations were run for each of the MODY type 1 through 5. The plots in Figure 7 show typical results from one 80 year simulation for of each of MODY types 1 through 5. Simulations are intended to demonstrate the role of network structure, particularly the feedback loops, on network dynamics. The time scales and relative reaction rates were set to give realistic output values, but are not intended to reproduce precise second-by-second enzyme kinetics (for model details, see Methods). Insulin production level in the model was used as a marker for disease severity. Overall, deficiencies in gene production higher up the cascade might be expected to cause more severe downstream effects on insulin production.

Although the model for this pathway is deterministic, the imposition of stochastic fluctuations on the expression level of the involved genes causes every simulation time series to be different and potentially to have a different outcome. As discussed previously, if the system has complex or nonlinear characteristics, small perturbations can result in large outcome changes. The random variation in each allele is independent of the other. When two alleles are present the total protein production rate is relatively high and random variation of each allele offsets the other. When a gene is haploinsufficient, random fluctuations are higher, sometimes sufficient to engage the positive feedback loop, resulting in disease onset. The mean age of disease onset, variability in age of onset and the mean insulin level (indicative of disease severity) were computed for each set of simulations. Known disease characteristics and simulation results are shown in Table 3.

**Figure 8:**
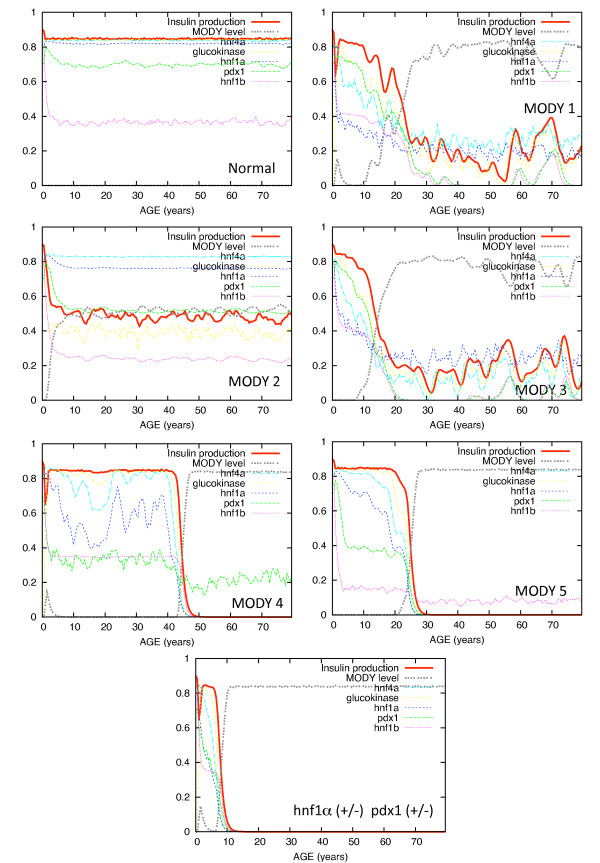
**MODY simulations**. Simulations of the MODY pathway shown in Figure 7 are shown for haploinsufficiency of hnf1a, glycokinase, hnf4a, Pd × 1 and hnf-1brespectively corresponding to MODY types 1 through 5.

The sample simulations in Figure 8 are representative of the behavior for that MODY type, though the age of disease onset may vary considerably depending on the type. MODY2, for example, exhibited relatively uniform dynamics for all simulations, with consistent early onset but mild hypoglycemia (indicated by moderately low insulin level). MODY4 simulations show the most variation in age of onset, which is not apparent in a single plot, but insulin levels were relatively uniform after disease onset. The MODY4 network appears to have two stable states, either no disease at all or very high disease level (low insulin production) and the transition from no disease to disease state occurs rapidly at a wide variety of ages. This is typical of complex system behavior, although we have not done the requisite mathematical analysis to establish true complex system characteristics [19].

In simulations, MODY2 (glycokinase insufficiency) appears to be milder than the other types and does not worsen with time, perhaps because glycokinase is not involved in any feedback loop. Onset of MODY2 in our models is consistently early, under age 10, which is consistent with known characteristics of MODY2 [42,44]. Once the disease is present, insulin deficiency in our model is relatively mild, also consistent with observed disease characteristics.

Insulin production in MODY types 1 and 3 is significantly reduced in simulations, but fluctuates without falling to zero even after disease onset. When either of the deficient gene products increases due to random fluctuations, insulin production increases slightly. The mean insulin level after disease onset in our models for MODY1 and MODY3 is significantly lower than for MODY2. Clinically, MODY 1 and MODY 3 have similar presentations. The differences in our simulations are primarily due to placement in the loop structure, although it is somewhat complicated by the double feedback from hnf4a to upstream components, hnf1a and pdx1. MODY 1 is quite rare, with only 13 families identified worldwide as of 2001 [42]. Our simulations suggest that the primary difference between these two forms is the age of onset and variability in the age of onset. The age of onset for MODY1 is earlier and less variable, starting at about 10 years, while MODY3 starts much later (average age 24 in our simulations) and the age of onset is much more variable. The mean insulin level after disease onset is higher in MODY1, suggesting possibly milder symptoms. It is important to emphasize that the ages and levels in our simulations should be interpreted in a relative sense to gain an understanding of how pathway dynamics might affect disease characteristics. The values and ages should not be considered precise predictions.

Deficiencies in pdx1 (MODY type 4) and hnf1bMODY type 5 might be expected to have the most severe effect on insulin production due to the direct feedback by insulin on gene expression or protein activity. MODY4 is a form of early onset type-II diabetes mellitus associated with a disruption in one allele of the Pdx1 or insulin promoter factor-1 (IPF1) gene [45]. Age of onset tends to be in early adulthood and symptoms are relatively mild. In one study, ketosis and other signs of severe insulin deficiency were lacking in patients with MODY4 [45]. In our simulations, MODY4 has the greatest variability in age of onset (11 years standard deviation) with an average age of 23 years. This is consistent with observed disease characteristics. However, our MODY4 model exhibits a switch-like behavior. Before disease onset, levels of insulin secretion are steady and relatively high. This state would manifest as mild or no disease symptoms. When random variation drops insulin low enough, the feedback loop engages and a change to the stable disease state occurs rapidly. Once in that state, insulin production is uniformly low. This latter condition is not consistent with observed symptoms, , suggesting that an additional element in the pathway may be missing that reduces the effect of Pdx1 deficiency. However, MODY4 is very rare, with studies based on a single extended family [42].

When Hnf-1b is haplodeficient (MODY5), low insulin production and disease onset appear occur consistently early in our simulations. This is not surprising, as is involved in a direct feedback loop between insulin secretion and Pdx1 at the top of the loop. Hnf-1b results in a distinct form of diabetes that is associated with a spectrum of related defects including renal cysts and internal defects in uterus and genitalia [42]. Clearly our model does not include interconnections with all of the other networks that regulate development of the renal system and other organs. Nevertheless, the key role played by Hnf-1b in the feedback loop of Figure 7 results in early and definitive disease onset in simulations when a haplodeficiency exists.

Finally, we note that [46] reported that heterozygosity in hnf1a (+/-) on a pdx1 (+/-) background produced strong decreases in insulin production in mice. This outcome is not surprising in light of the above results, as a deficiency in each of these proteins will lead to the disease state. Simulation results, shown in Figure 8, are as expected: the double haplodeficiency causes early and severe restriction of insulin production.

## Discussion

As we have discussed elsewhere [47], network structure is a critical determinant of network dynamics and dynamics is often not apparent from structure without simulation. Our purpose here was to examine simple representations of positive feedback loop structures and their response to noisy inputs at the top of a signaling cascade because these mechanisms appear to be involved in many haploinsufficiency diseases. Our basic theory is that stochastic gene variation in proteins that are involved in positive feedback loops can cause network dynamics that may be responsible for some haploinsufficiency diseases. A catalog of networks with feedback loops was presented and the simulation results for each network was constructed to illustrate the dynamics of the network structure, which is not always evident from the visual topology of the network. These simulations are intended to give insight into the mechanisms that cause more severe disease in some forms or mild, reversible symptoms in others.

As noted in the stochastic simulations in Figure 5, the magnitude of expression variation and the time scale over which it varies are both important parameters. Even small dips in expression, if they persist long enough, may cause significant drift of key protein concentrations from required minimum levels and allow a disease state to occur. If a positive feedback loop is involved, that may be sufficient to trigger a transition to a permanent disease condition.

Polycystic kidney disease involves a number of complex interacting pathways that undoubtedly add to the complexity of this disease. Nevertheless, the simple model presented here seems to be able to capture some of the essential features of this disease. In particular, the feedback loop involving TNF-α is a critical switch that controls the change from normal function to the sustained growth of cysts once the disease has set in. The model results suggest that drugs that inhibit the feedback loops are likely to be effective in reducing the disease severity.

Differences in MODY characteristics that were found in simulations reflect many of the differences in the actual phenotypes. The general conclusions that can be drawn from the simulation in Figure 8 are that when network components are higher up the cascade of events in a process, the more profound their effect on system dynamics. Several important disease characteristics might be surmised from the simulations alone. First, haplodeficiency of Pdx1 and hnf-1b are likely to cause earlier onset and greater insulin need than haplodeficiency of hnf4a and glycokinase. Glycokinase deficiency (MODY 2) is likely to not worsen with age in this model because there is no feedback loop to amplify stochastic reductions in glycokinase. An adaptation has been observed in mice with glucokinase related MODY (type 2). Experiments suggest that mild hyperglycemia leads to increased expression of the functioning glucokinase gene, thus limiting the severity of the defect in glucose-stimulated insulin secretion [48]. Our simulations suggest that this increased expression is not required to explain the limited severity of MODY type 2 symptoms. The mildness of MODY 2 rather seems to result from glycokinase not being involved in any feedback loops in the MODY pathway. Haploinsufficiency in the other proteins that have feedback loops can result in definite decreases in insulin production at specific ages when the feedback loop is engaged. However, although insulin level decline is not as severe even when the disease is present, the switch from no disease to a disease state appears to be sensitive in our simulations. The result is that disease onset tends to be consistently early. The effect of glycokinase deficiency is apparently felt in the loop dynamics and the switch to the disease state requires only small stochastic perturbations to engage.

## Conclusions

In summary, results presented above show that the onset and variability of haploinsufficient diseases may be intimately linked to the structure and dynamics of the regulatory networks underlying the processes affected in these diseases, which should be taken into account in designing approaches for medical intervention. The primary purpose of the computational models is to enable the dynamics of a complex pathway to be explored, as dynamics are often not apparent from the appearance of the network structure alone. While much more detailed modeling studies are required for realistic drug target testing, analysis of regulatory networks through simple model simulation are useful for assessing the disease mechanism and possibly more precise treatment options by targeting key parts of a complex pathway.

## Methods

Simulations were run with each of these networks using the Bionet simulator [26]. The models that are derived from the drawings in Figure 1 all have a production and decay reaction for each of the three components. Degradation is a constant first order reaction for all components and is not regulated. Production or expression occurs at a rate that is modulated by feedbacks as shown in the diagram. To characterize the dynamic response of each of the networks, simulations were run with noise sources added to the production rates of either X or Y separately, or to both.

Variation in gene expression was simulated with zero-mean Gaussian noise, with standard deviation of 1.0. Since Gaussian noise can have positive or negative values, the production rates will increase or decrease when Gaussian noise is added. Our intention was to simulate the effects of stochastic gene expression using Gaussian noise. As discussed previously, gene expression can vary significantly on time scales of hours to days. Protein concentrations will vary somewhat more slowly, as the protein concentration is a cumulative effect of gene production. In our models, adding noise to the production rate simulates this effect.

For our simulations, setting appropriate reaction rates is essential, including production and activation or inhibition effects. If production of a critical gene product is set too high, then haploinsufficiency is irrelevant. In addition, a minimum threshold level for disease growth must be assumed. This assumption derives naturally from the switch-like dynamics of biological positive feedback loops. Below a certain threshold, no reaction occurs, while above that threshold the reaction is rapid. Otherwise, disease growth will always be present to some degree, except in the trivial case where all reactants have zero concentration. As much as possible, realistic values for rates and timescales are chosen for this study. Concentrations shown are normalized to the range 0 to 1. For more detailed model input in the Bionet simulator, see Model input files for execution in the bionet simulator, available online at [25].

## Authors' contributions

WJB designed computer models, carried out simulations of all feedback networks and created computer models of diseases in this study. RL conceived of the study, participated in model design and evaluation of simulation results. Both authors participated in writing the manuscript and have read and approve the final manuscript.
